# Stress-Induced Cardiomyopathy Following Elective Lumbar Disc Nucleotomy: Multimodal Cardiac Evaluation With 2D-STE and D-SPECT in a Patient With Severe Cardiac Magnetic Resonance (CMR) Contraindication

**DOI:** 10.7759/cureus.115227

**Published:** 2026-08-26

**Authors:** Ying Wei, Xuebin Wang, Peng Su, Ling Xing, Jiyang Song

**Affiliations:** 1 Department of Cardiology, Gansu Provincial Hospital, Lanzhou, CHN; 2 Department of Clinical Medicine, Qinghai University Medical College, Xining, CHN

**Keywords:** 2d-ste, d-spect, lumbar nucleotomy, perioperative cardiac injury, sacubitril/valsartan, stress-induced cardiomyopathy, takotsubo syndrome

## Abstract

Minimally invasive lumbar spinal surgery can precipitate rare perioperative Takotsubo syndrome (stress-induced cardiomyopathy, SIC), a condition frequently misdiagnosed as acute myocardial infarction. Cardiac magnetic resonance (CMR) serves as a valuable diagnostic tool for the multimodal evaluation of Takotsubo syndrome, helping exclude alternative etiologies such as myocarditis. Diagnosis of Takotsubo syndrome relies on comprehensive integration of clinical manifestations, ventricular imaging, coronary angiography, electrocardiography, and cardiac biomarkers, rather than depending on CMR alone. We herein report the case of a 66-year-old postmenopausal woman without conventional cardiovascular risk factors who developed Takotsubo syndrome shortly after elective lumbar discectomy. Obstructive coronary artery lesions were ruled out by coronary angiography. Owing to the patient’s claustrophobia, we implemented a combined imaging protocol of two-dimensional speckle-tracking echocardiography (2D-STE) and dynamic single‑photon emission computed tomography (D-SPECT), which verified reversible myocardial dysfunction. The patient received guideline-directed medical therapy consisting of sacubitril/valsartan, beta-blockers, mineralocorticoid receptor antagonists, and a sodium‑glucose cotransporter 2 (SGLT2) inhibitor. Cardiac biomarkers returned to normal levels on postoperative day 11, and myocardial strain parameters showed substantial improvement at the eight-week outpatient follow-up. This case underscores the need for clinical vigilance for Takotsubo syndrome after spinal surgery. In this individual patient with CMR contraindications, combined 2D‑STE and D‑SPECT provided complementary supportive diagnostic information. Further studies are required to validate the general utility of this multimodal imaging approach.

## Introduction

Minimally invasive lumbar spinal surgery is widely performed for degenerative lumbar disc disease, with generally favorable perioperative safety profiles. Nevertheless, rare life‑threatening cardiac complications may develop in the early postoperative period, which are easily misdiagnosed as primary coronary artery events and delay targeted intervention. Stress‑induced cardiomyopathy (SIC), also termed Takotsubo syndrome, is triggered by intense physical or emotional stress, characterized by transient left ventricular systolic dysfunction without obstructive coronary lesions [1]. The international expert consensus systematically summarized the core clinical features, diagnostic criteria, and underlying pathophysiological mechanisms of this disease [1]. The Heart Failure Association of the European Society of Cardiology also released a position statement to elaborate the latest research progress of Takotsubo syndrome [2]. Its incidence after orthopedic spinal procedures remains underrecognized, and standardized diagnostic workflows are lacking for patients who cannot undergo cardiac magnetic resonance (CMR) due to severe claustrophobia [3,4]. Multiple case reports have documented Takotsubo cardiomyopathy as an unexpected perioperative complication following spinal operations [3-6].

Two‑dimensional speckle‑tracking echocardiography (2D‑STE) provides quantitative evaluation of myocardial strain, which can dynamically reflect the time course of left ventricular functional recovery in Takotsubo syndrome [7]. Layer‑specific myocardial deformation can be accurately quantified via serial 2D‑STE to identify subtle systolic dysfunction [8]. CMR serves as a valuable diagnostic tool for the multimodal evaluation of Takotsubo syndrome, helping exclude alternative etiologies such as myocarditis, yet a proportion of patients fail to tolerate the confined scanning environment [9]. A study published in the Journal of Cardiovascular Magnetic Resonance described the key diagnostic role of CMR for detecting characteristic myocardial edema in Takotsubo syndrome [9]. Nuclear myocardial perfusion imaging via dynamic single‑photon emission computed tomography (D‑SPECT) offers complementary structural and functional cardiac data. Combined application of these two imaging modalities may deliver supplementary diagnostic information for patients when CMR cannot be performed. Sacubitril/valsartan and other heart failure medications are well‑established therapies for reduced left ventricular ejection fraction (LVEF). A clinical cohort research confirmed sacubitril/valsartan could suppress myocardial inflammation and fibrosis in Takotsubo‑like myocardial injury models [10]. JACC issued practical guidance for standardized clinical application of sacubitril/valsartan in heart failure patients [11], but relevant practical case descriptions after lumbar surgery remain scarce [4].

This report presents a postmenopausal female patient without traditional cardiovascular risk factors who developed typical SIC immediately after elective lumbar disc nucleotomy. We describe the multimodal cardiac evaluation protocol combining 2D‑STE and D‑SPECT, as well as guideline‑directed individualized medical management. The case aims to raise clinicians’ awareness of perioperative Takotsubo syndrome following spinal surgery and provide a practical imaging scheme for patients ineligible for CMR scanning.

## Case presentation

**Figure 1:**
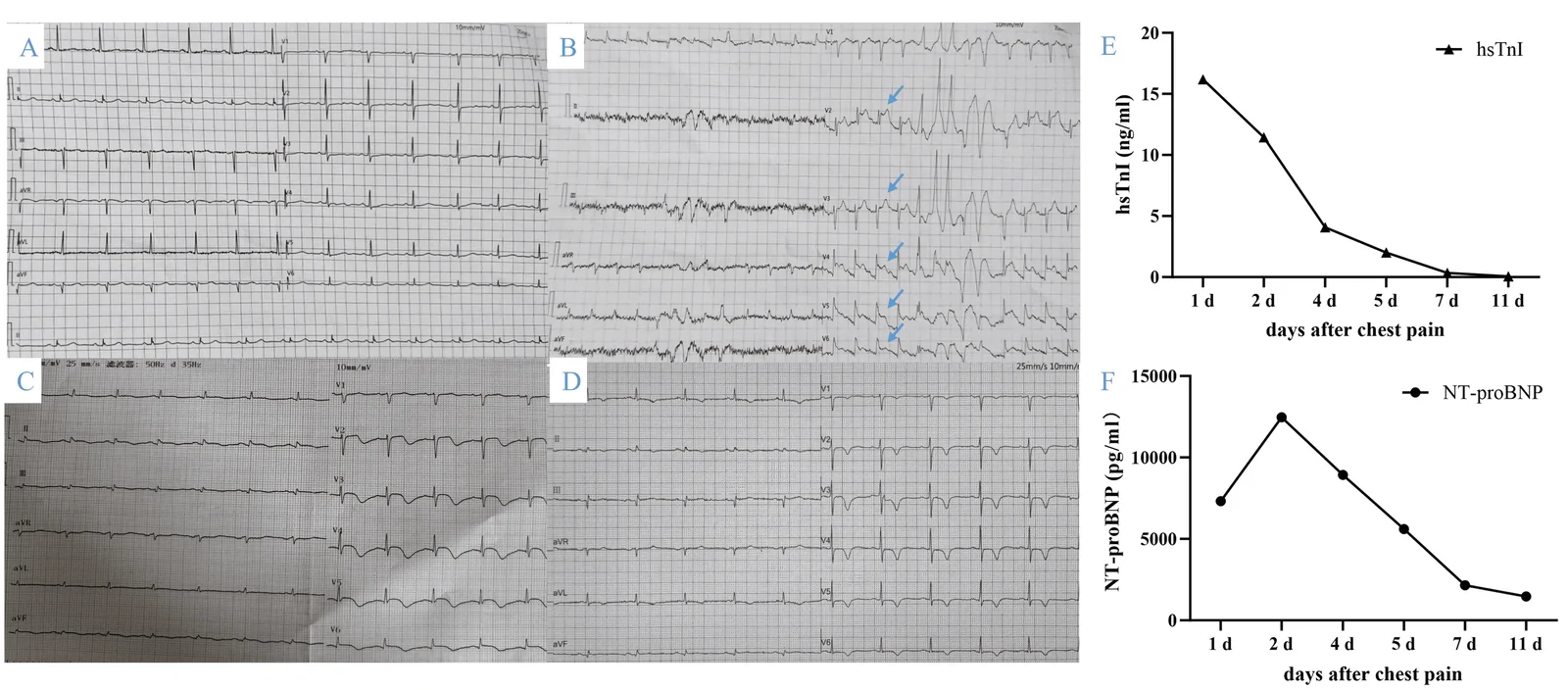
Dynamic changes in electrocardiograms and cardiac biomarkers (A-D) Dynamic serial changes in electrocardiograms over time. Panel A shows a normal electrocardiogram on admission. Panel B shows an electrocardiogram during chest pain, with straight digital arrows pointing to elevated ST segments in leads V2-V6. Panels C and D display electrocardiographic changes during recovery. Panels E and F demonstrate serial changes in high‑sensitivity cardiac troponin I and NT‑proBNP, showing acute elevation followed by complete normalization.

A 66-year-old postmenopausal woman presented with a three-month history of intractable low back pain and right lower limb radiating pain that failed conservative treatment. The patient’s body mass index (BMI) was 27.34 kg/m². Baseline blood pressure was normal. There was no history of psychiatric anxiety disorder. The patient had no conventional cardiovascular risk factors, including hypertension, diabetes mellitus, hyperlipidemia, smoking, or alcohol consumption. Preoperative routine electrocardiography and transthoracic echocardiography demonstrated completely normal cardiac structure and systolic function (Figure 1A), with no preoperative cardiovascular medications administered.

The patient underwent elective lumbar disc nucleotomy under general anesthesia at an outside county‑level hospital. No complete intraoperative or anesthetic records were transferred upon hospital transfer; therefore, detailed anesthetic agents and continuous real‑time hemodynamic data are unavailable for retrospective analysis. Thirty minutes after anesthesia recovery, the patient developed acute severe chest pain and progressive dyspnea. Physical vital sign monitoring revealed sinus tachycardia at 110 beats per minute. Emergency 12‑lead electrocardiography showed significant ST‑segment elevation in leads V2‑V6, highly suggestive of acute myocardial ischemic injury (Figure 1B). Subsequent laboratory tests showed elevated high‑sensitivity troponin‑I (hs‑TnI) of 16.1933 ng/mL (reference range < 0.04 ng/mL) and NT‑proBNP of 7320 pg/mL (reference range < 300 pg/mL). Bedside emergency echocardiography detected obvious regional wall motion abnormalities, a reduced LVEF of 35%, and mild mitral regurgitation.

**Figure 2:**
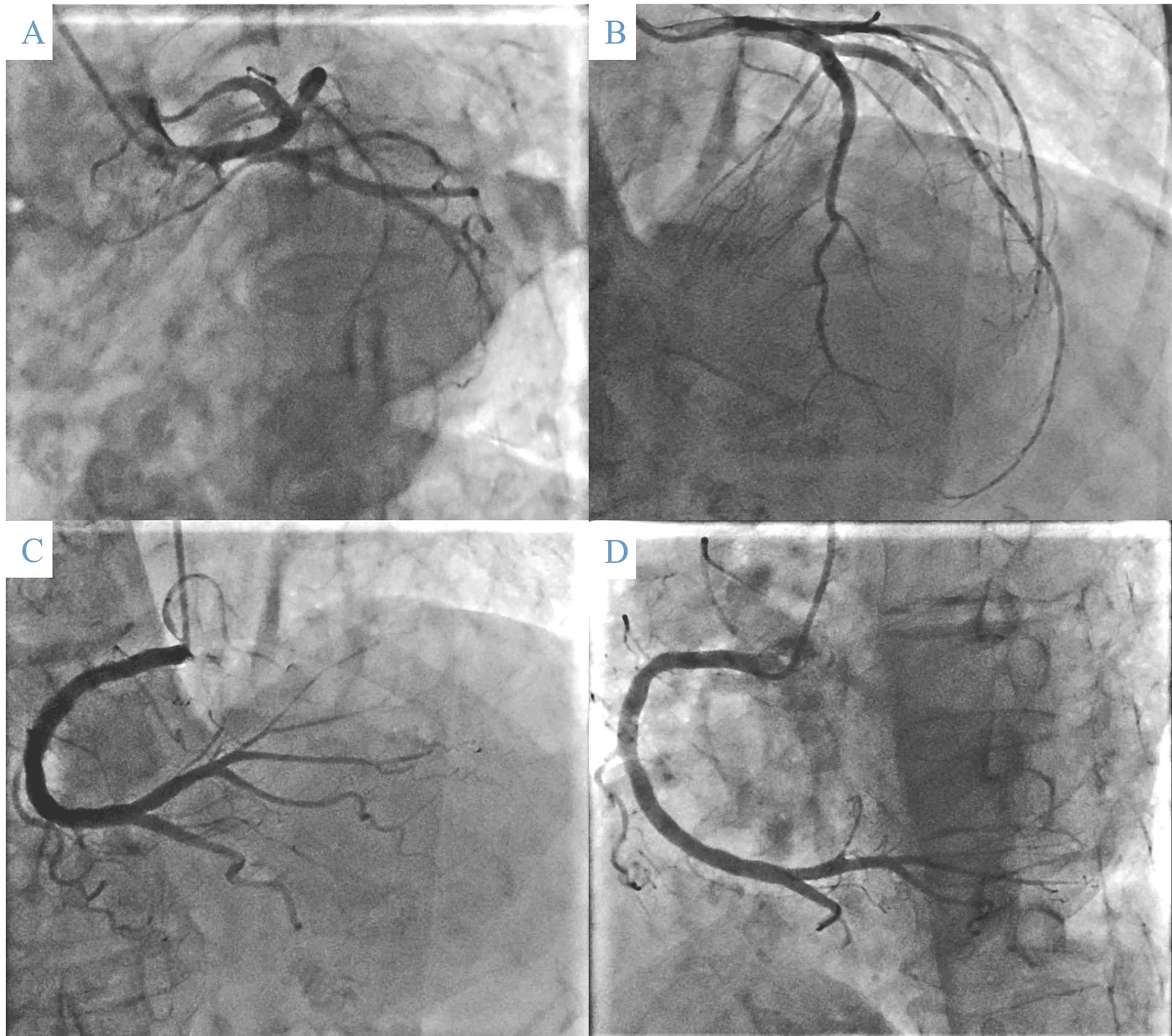
Coronary angiography findings (A, B) Left coronary artery. (C, D) Right coronary artery. All views demonstrate patent coronary arteries, without significant stenosis, occlusion, or coronary vasospasm during the acute chest pain episode.

Urgent coronary angiography was performed to exclude acute myocardial infarction, demonstrating completely patent coronary arteries with TIMI 3 blood flow in both left and right coronary systems, without obstructive stenosis, atherosclerotic lesions, or evidence of coronary vasospasm at the time of acute chest pain onset (Figure 2). The patient had no fever throughout the clinical course, and cardiac biomarkers demonstrated a transient elevation, which is inconsistent with the typical pattern of myocarditis. Nevertheless, because CMR could not be performed due to patient claustrophobia, rare atypical myocarditis and other myocardial infarction with non-obstructive coronary arteries (MINOCA)‑related myocardial injury cannot be definitively ruled out. Takotsubo syndrome remains the most probable diagnosis based on composite clinical, electrocardiographic, biomarker, angiographic, and multimodal imaging findings.

**Figure 3:**
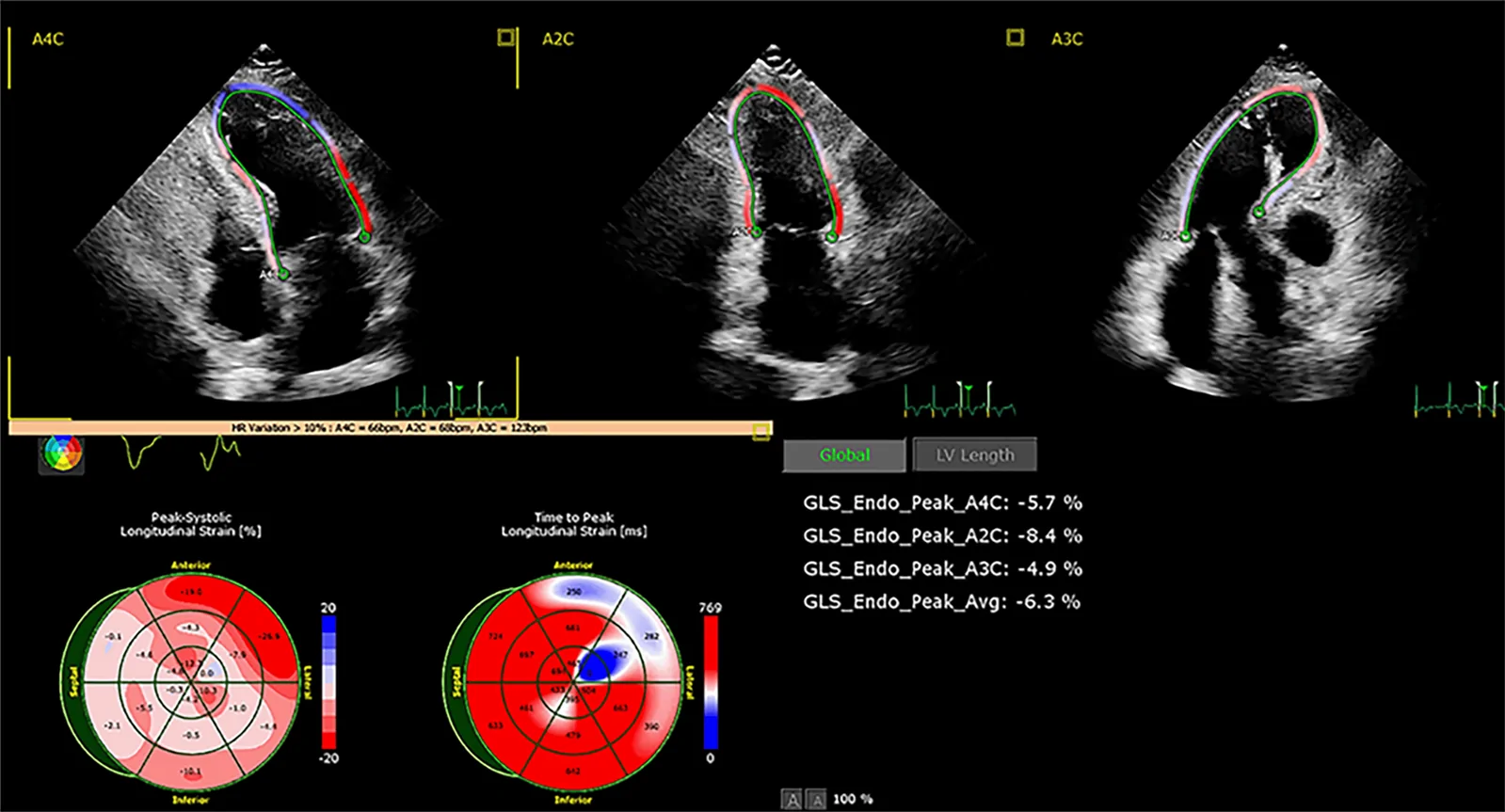
Acute myocardial strain abnormalities detected by two-dimensional speckle-tracking echocardiography (2D‑STE) Acute‑phase 2D‑STE demonstrating typical apical ballooning and severely reduced left ventricular global longitudinal strain.

**Figure 4:**
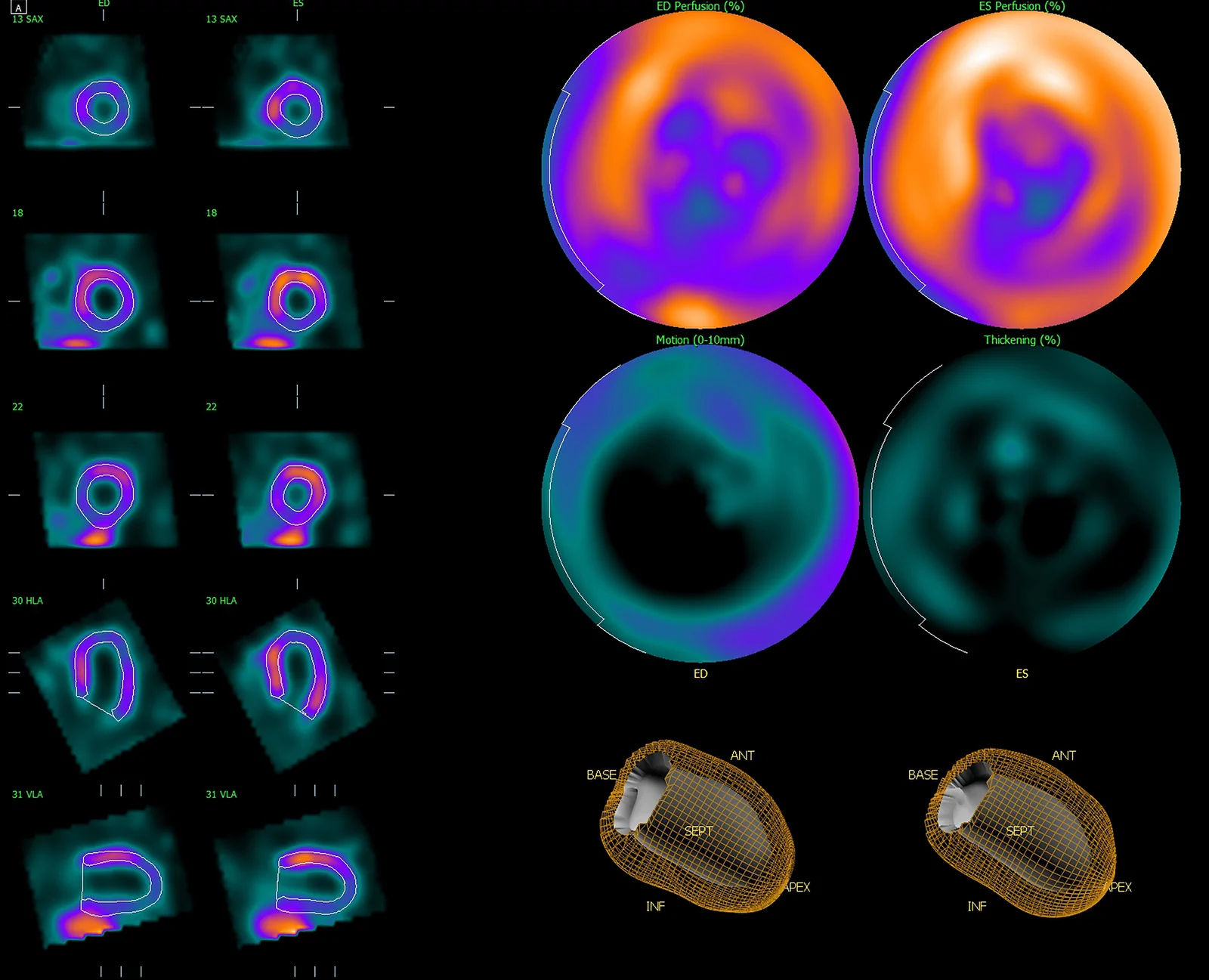
Patchy myocardial tracer uptake abnormalities demonstrated on dynamic single‑photon emission computed tomography (D‑SPECT) myocardial perfusion imaging D‑SPECT myocardial perfusion imaging showing characteristic patchy tracer uptake abnormalities in the apical and mid‑ventricular segments.

Considering the patient's severe claustrophobia, CMR imaging could not be implemented for confirmatory diagnosis. Emergency 2D-STE identified typical apical ballooning deformity and significantly decreased global longitudinal strain (GLS) of -6.3%, consistent with the characteristic manifestations of SIC (Figure 3). Rest‑gated myocardial‑perfusion D‑SPECT was performed after intravenous injection of 20 mCi technetium‑99m‑MIBI, with imaging acquired 1.5 hours later. The left ventricular cavity was non‑dilated. Heterogeneous myocardial tracer uptake was seen in the apex, inferior wall, and apical‑middle segments of the lateral wall. QGS software semiquantitative analysis showed end-diastolic volume (EDV) 76 mL, end-systolic volume (ESV) 61 mL, and LVEF 20%. Attenuation artifacts were unlikely based on gated reconstruction, angiographic, and echocardiographic correlation. Without quantitative myocardial flow data, these patchy perfusion abnormalities cannot be ascribed to definite coronary microvascular dysfunction (Figure 4). Combined with normal coronary angiography, transient biomarker elevation, and typical wall motion pattern, these findings are most consistent with Takotsubo syndrome rather than ischemic injury [8].

Standard guideline-directed medical therapy was promptly initiated, including beta-blockers, sodium-glucose cotransporter 2 (SGLT2) inhibitors, and mineralocorticoid receptor antagonists [12]. Serial monitoring of cardiac biomarkers and electrocardiographic changes was performed during hospitalization. Follow-up examinations at one week and eight weeks postoperatively showed a gradual resolution of abnormal ST-segment changes on electrocardiography (Figure 1C, 1D). All elevated cardiac biomarkers returned to normal levels by postoperative day 11 (Figure 1E, 1F).

**Figure 5:**
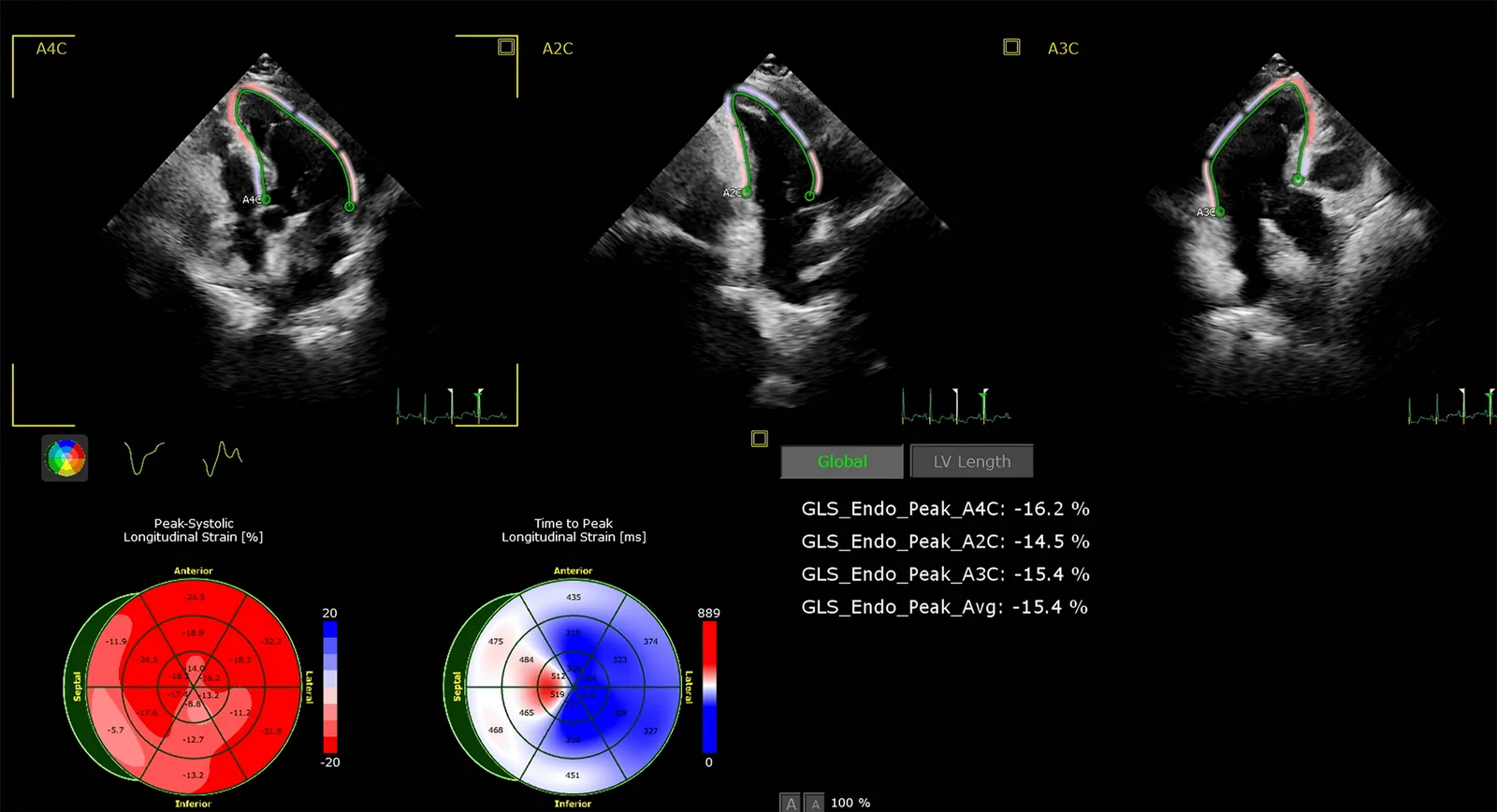
Eight-week follow-up two-dimensional speckle-tracking echocardiography (2D-STE) showing complete recovery of left ventricular longitudinal strain and normal cardiac function

The eight-week outpatient follow-up 2D-STE confirmed complete recovery of left ventricular systolic function, with LVEF improved to 54% and GLS significantly restored to -15.4% (Figure 5).

The patient was treated with sacubitril/valsartan, SGLT2 inhibitors, and mineralocorticoid receptor antagonists during the period of reduced LVEF. All medications were gradually discontinued after LVEF fully recovered. The patient remained asymptomatic with stable daily activity throughout follow-up, without recurrent cardiac symptoms.

Written informed consent was obtained from the patient for publication of de-identified clinical data and all imaging findings. All personally identifiable information has been thoroughly removed from the manuscript to protect patient privacy.

## Discussion

SIC, namely Takotsubo syndrome, is a life‑threatening perioperative complication that can be triggered by the surgical stress of minimally invasive lumbar spinal procedures [4]. Characterized by acute and reversible left ventricular dysfunction, SIC presents with clinical symptoms, electrocardiographic changes, and elevated cardiac biomarkers that highly mimic acute myocardial infarction, easily leading to clinical misdiagnosis and delayed intervention [13,14]. Even patients without traditional cardiovascular risk factors may develop SIC under the intense physiological stress of spinal surgery, reminding orthopedic and anesthesiology clinicians to maintain high postoperative vigilance for this rare complication.

Coronary angiography is the essential examination to rule out obstructive acute coronary events, while CMR serves as the diagnostic gold standard for SIC by detecting specific myocardial edema and injury [9]. However, severe claustrophobia‑induced CMR intolerance creates a common clinical diagnostic dilemma, leaving a subset of patients without standardized confirmatory diagnostic tools. For such CMR‑ineligible patients, our multimodal imaging protocol combining 2D‑STE and D‑SPECT provided comprehensive supportive cardiac evaluation in this case. 2D‑STE quantified typical myocardial strain abnormalities and apical‑predominant systolic dysfunction [7], while D‑SPECT demonstrated patchy myocardial tracer uptake abnormalities in this patient. Without quantitative myocardial blood flow or flow reserve data, definite microvascular dysfunction could not be confirmed. This combined imaging strategy offered complementary diagnostic information for this individual patient when CMR could not be performed.

Early initiation of guideline‑directed medical therapy is recommended for patients presenting with left ventricular systolic dysfunction secondary to SIC [11,12]. Sacubitril/valsartan, SGLT2 inhibitors, and mineralocorticoid receptor antagonists are well‑established therapies for heart failure with reduced ejection fraction. Treatment decisions in the present case followed general heart‑failure principles, rather than Takotsubo‑specific guideline recommendations. Spontaneous recovery of left ventricular function is a well‑recognized hallmark of SIC. Although cardiac biomarkers normalized within 11 days and full left ventricular systolic recovery was observed at the eight‑week follow‑up, we cannot definitively attribute this favorable clinical course solely to pharmacotherapy, as the natural self‑limiting course of Takotsubo syndrome may also account for this improvement [15]. This case illustrates the well‑known reversibility of SIC under standardized clinical management.

Existing clinical studies mainly focus on SIC induced by emotional stress or internal medical diseases, while reports focusing on spinal surgery‑related SIC in patients with CMR contraindications are extremely limited [3,6,16]. The present case supplements valuable real‑world clinical evidence for this special patient population. Our observations illustrate the potential auxiliary value of combined 2D‑STE and D‑SPECT for suspected postoperative SIC among CMR‑intolerant patients, but do not validate it as a generalized alternative diagnostic pathway. Timely diagnosis and standardized management may facilitate favorable clinical outcomes, providing a practical reference for the early recognition and comprehensive management of perioperative SIC in spinal surgery patients.

Limitations

First, the index lumbar spine surgery and general anesthesia were performed at an outside county‑level hospital. Complete intraoperative and anesthesia‑related medical records were not transferred upon patient admission to our emergency department. Thus, detailed anesthetic agents, anesthesia mode, intraoperative hemodynamic events, surgery‑anesthesia duration, fluid management, and intraoperative adverse events cannot be retrospectively analyzed.

Second, preoperative troponin and NT‑proBNP were not tested at the external hospital; true preoperative baseline cardiac biomarker values are unavailable. Cardiac magnetic resonance imaging could not be performed due to the patient’s claustrophobia, so rare atypical myocarditis and other MINOCA‑related myocardial injury cannot be definitively ruled out.

Third, observations from this single case cannot establish the general diagnostic accuracy of combined 2D‑STE and D‑SPECT. This dual‑modality imaging only provided complementary supportive diagnostic information. Quantitative myocardial‑flow‑reserve data were also absent to confirm microvascular dysfunction.

Fourth, spontaneous recovery is a well‑recognized hallmark of Takotsubo syndrome. We cannot distinguish the contribution of heart failure‑directed pharmacotherapy from the natural disease course. Treatment was guided by general HFrEF clinical recommendations.

## Conclusions

Perioperative Takotsubo syndrome represents a rare but potentially severe complication of minimally invasive lumbar spinal surgery, which may occur even in patients without conventional cardiovascular risk factors and is easily misdiagnosed as acute myocardial infarction. In this individual case with claustrophobia precluding CMR examination, combined 2D‑STE and D‑SPECT revealed reversible myocardial strain changes and patchy myocardial tracer uptake abnormalities and provided supportive information for clinical work‑up. Early guideline‑directed medical therapy including beta‑blockers, SGLT2 inhibitors, and mineralocorticoid receptor antagonists was administered according to general heart failure principles. Complete resolution of myocardial injury and full recovery of left ventricular function were observed during follow‑up. This case highlights the necessity of rigorous perioperative cardiac monitoring for spinal surgery patients. It also illustrates the potential clinical value of dual‑modality imaging as a feasible auxiliary diagnostic approach for Takotsubo syndrome when CMR is unavailable, providing a single‑case reference for the early recognition and standardized management of perioperative Takotsubo syndrome.
